# Anesthetic Management of Cervicomedullary Decompression in a Child With Down Syndrome: A Case Report

**DOI:** 10.1002/pdi3.70078

**Published:** 2026-09-21

**Authors:** Muhammad Saad, Marium Amjad

**Affiliations:** ^1^ Department of Anesthesiology Dow University of Health Sciences Karachi Pakistan

**Keywords:** airway management, cervical spine instability, down syndrome, general anesthesia, pediatric neurosurgery

## 1 Introduction

Down syndrome (trisomy 21) is the most common chromosomal abnormality, occurring in approximately 1.5 per 1000 live births [1]. It affects multiple organ systems, including the cardiovascular, respiratory, musculoskeletal, and nervous systems, all of which have anesthetic implications. Characteristic features such as midface hypoplasia, macroglossia, subglottic stenosis, and hypotonia contribute to potential airway difficulties. Moreover, cervical spine instability, particularly atlanto‐axial subluxation, is observed in nearly one‐third of patients, increasing the risk of spinal cord injury during anesthesia or surgery [2]. These patients also frequently have congenital heart disease, most commonly atrioventricular septal defects, which can further complicate perioperative management.

Perioperative adverse events in Down syndrome are well‐documented, with airway obstruction, bradycardia, postintubation croup, bronchospasm, and postoperative respiratory failure being particularly common [3]. Such patients require detailed preoperative assessment and tailored anesthetic strategies that address individual comorbidities. This report describes the anesthetic management of a child with Down syndrome undergoing cervicomedullary decompression and occipito‐cervical fusion, emphasizing the planning and precautions necessary to minimize risks and optimize safety.

## 2 Case Description

A 13‐year‐old boy weighing 50 kg, diagnosed with Down syndrome, presented with progressive weakness of all four limbs and gait unsteadiness for six months. There was no prior surgical history or record of cardiac or respiratory illness. According to his parents, he snored mildly but had no witnessed apnea or daytime somnolence. Neurological examination revealed spasticity in both lower limbs, upgoing plantar responses, and reduced power in both upper limbs (2/5). The patient's gag reflex was unaffected, and both the cervical spine CT scan and MRI of the brain (Figure 1) revealed compression of the cervicomedullary junction due to a craniovertebral junction anomaly. Echocardiography revealed a small, hemodynamically insignificant atrial septal defect with preserved biventricular function.

**FIGURE 1:**
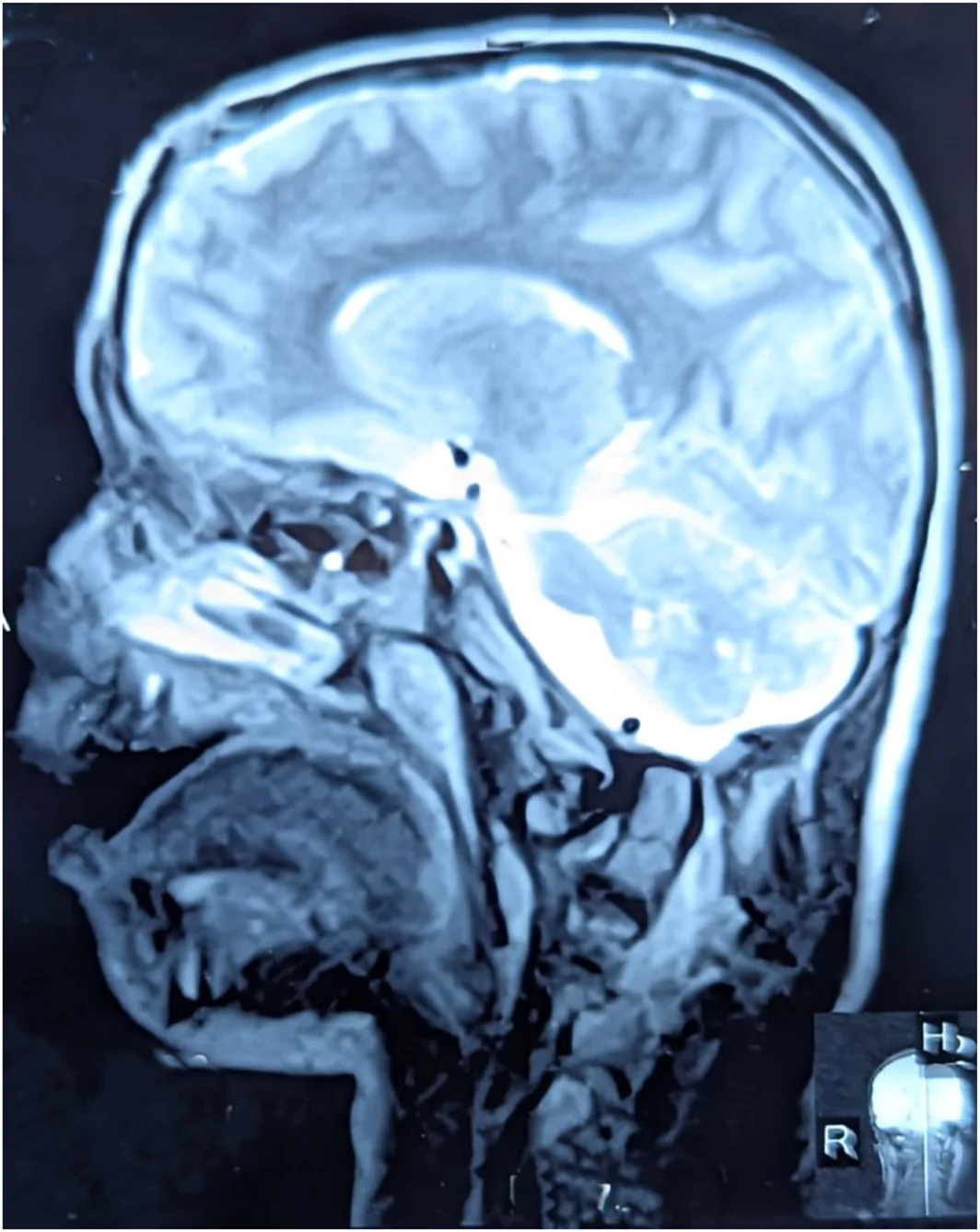
Magnetic resonance imaging (MRI) of the brain showing compression of the cervicomedullary junction due to a craniovertebral junction anomaly.

The surgical team planned posterior decompression with occipito‐cervical arthrodesis. Given the risks associated with trisomy 21—difficult airway, cervical instability, and cardiac anomalies—the anesthetic approach was designed to maintain cervical neutrality, ensure a secured airway with minimal manipulation, and prevent hemodynamic fluctuations. Premedication included intravenous midazolam 0.5 mg for anxiolysis and dexamethasone 4 mg to reduce postoperative edema and nausea.

Standard ASA monitoring (ECG, non‐invasive blood pressure, pulse oximetry, end‐tidal CO_2_, and temperature) was established. Induction was achieved with sevoflurane in oxygen via a Mapleson A circuit while maintaining spontaneous ventilation. After confirming mask ventilation, a left‐hand 18‐G intravenous cannula was inserted, and a right radial arterial line was secured aseptically for invasive blood pressure monitoring. Intravenous nalbuphine 5 mg, propofol 100 mg, and atracurium 25 mg were administered sequentially once spontaneous breathing ceased. Fentanyl, sufentanil, and morphine were not available at our hospital. Therefore, nalbuphine was used at a standard dose of 0.1 mg/kg.

Tracheal intubation was performed using a C‐MAC video laryngoscope (size 3 blade) with manual inline stabilization to avoid cervical movement. An oral 6.0 mm cuffed endotracheal tube (Medis Medical, external diameter 8.8 mm) was placed without difficulty. The Mallampati score was III. Nasotracheal intubation was avoided to minimize neck manipulation. Tube position was confirmed with bilateral auscultation and capnography.

The patient was positioned prone using a coordinated three‐person log‐roll technique, maintaining the head and neck in a neutral alignment. The head was supported on a padded horseshoe headrest, with all pressure points and the eyes carefully protected. The abdomen was allowed to hang free to prevent diaphragmatic compression. Ventilation was pressure‐controlled (tidal volume 6–8 mL/kg, respiratory rate 18/min, PEEP 3 cm H_2_O, FiO_2_ 0.5) to maintain normocapnia (EtCO_2_ 35–40 mmHg). Intraoperative blood gases confirmed stable ventilation and oxygenation.

Anesthesia was maintained with sevoflurane (1.5%–2%) in oxygen and air. Neuromuscular blockade was assessed clinically, as formal train‐of‐four monitoring was unavailable. Pre‐operatively, the patient had a hemoglobin level of 12 gm/dL, and the total blood loss was approximately 200 mL. Post‐operative hemoglobin levels were found to be 11 gm/dL, so no transfusion was done. Intraoperative fluids consisted of 1000 mL of normal saline. The surgery lasted 180 minutes and involved posterior decompressive laminectomy with occipito‐cervical fusion. No hemodynamic instability occurred.

At the end of surgery, neuromuscular blockade was reversed with neostigmine 2.5 mg and glycopyrrolate 0.4 mg IV. The child was extubated awake after demonstrating adequate spontaneous breathing, airway reflexes, and purposeful movement. Oxygen was administered via a facemask post‐extubation. He remained hemodynamically stable and was transferred to the post‐anesthesia care unit for close observation, followed by admission to the neurosurgical high‐dependency unit. Analgesia was provided with paracetamol 15 mg/kg IV every six hours for the first 24 hours. The postoperative period was uneventful, and the patient was discharged on the seventh postoperative day with full neurological recovery.

## 3 Discussion

Down syndrome poses significant anesthetic challenges due to multisystem involvement. Airway anomalies, macroglossia, and subglottic narrowing predispose to difficult ventilation and intubation. Atlanto‐axial instability further increases the risk of spinal cord compression during airway manipulation [2]. In our case, the use of a video laryngoscope and manual stabilization minimized cervical motion during intubation, in accordance with best practice recommendations [4]. Preoperative echocardiography was invaluable to identify potential cardiac risks that could influence anesthetic planning.

Sevoflurane was chosen for induction to maintain spontaneous respiration until airway control was achieved, offering safety in patients at risk of airway compromise. Although desflurane allows quicker emergence, it may provoke airway irritation in children [5]. Careful patient positioning during prone neurosurgical procedures is equally critical. We employed a coordinated log‐roll maneuver with neutral neck alignment, protecting all pressure points and eyes, consistent with established neurosurgical safety protocol.

Pressure‐controlled ventilation allowed adequate gas exchange while minimizing barotrauma risk. Maintaining normocapnia and normothermia was essential for cerebral protection and hemodynamic stability. Extubation in Down syndrome requires caution because of hypotonia and potential postoperative airway collapse; performing it to ensure airway safety when fully awake. Continuous postoperative monitoring in a high‐dependency setting minimized the risk of respiratory complications.

This case reinforces critical principles for managing children with Down syndrome undergoing complex neurosurgical interventions: comprehensive preoperative evaluation, maintenance of cervical stability, controlled airway manipulation, and vigilant postoperative monitoring. When executed systematically within a multidisciplinary framework, these measures allow safe and successful outcomes even in high‐risk craniovertebral procedures.

## 4 Conclusion

Anesthetic management of children with Down syndrome demands detailed preoperative assessment, careful airway planning, and interdisciplinary coordination. Sevoflurane induction with video‐assisted intubation under cervical stabilization, combined with invasive monitoring and controlled ventilation, ensures perioperative safety. Attention to positioning, pressure protection, and awake extubation can further optimize outcomes. With individualized planning and vigilant monitoring, even complex neurosurgical procedures can be performed safely in this patient population.

## Author Contributions

**Muhammad Saad:** Literature search and manuscript writing. **Marium Amjad:** Critical revision and drafting.

## Funding

The authors have nothing to report.

## Ethics Statement

Institutional Review Board approval was not required for this single‐patient case, as all identifying information has been anonymized and no experiment was done.

## Consent

Informed consent was obtained from the patient's parents for publication of the clinical details and images.

## Conflicts of Interest

The authors declare no conflicts of interest.

## Data Availability

Data sharing is not applicable to this article, as no datasets were generated or analyzed during the current study.
